# Supplementary material for: Diverse silent chromatin states modulate genome compartmentalization and loop extrusion barriers
Source: Nat Struct Mol Biol. 2022 Dec 22;30(1):38–51. doi: 10.1038/s41594-022-00892-7 (PMC9851908; doi:10.1038/s41594-022-00892-7)
Supplement: Supplementary file 2 — Reporting Summary [file 41594_2022_892_MOESM2_ESM.pdf]

nature portfolio

Corresponding author(s): Dr. Job Dekker, Dr. George Spracklin

Last updated by author(s): Aug 27, 2021

Reporting Summary

Nature Portfolio wishes to improve the reproducibility of the work that we publish. This form provides structure for consistency and transparency in reporting. For further information on Nature Portfolio policies, see our Editorial Policies and the Editorial Policy Checklist.

Statistics

For all statistical analyses, confirm that the following items are present in the figure legend, table legend, main text, or Methods section.

n/a	Confirmed	
☐	☒	The exact sample size (n) for each experimental group/condition, given as a discrete number and unit of measurement
☐	☒	A statement on whether measurements were taken from distinct samples or whether the same sample was measured repeatedly
☒	☐	The statistical test(s) used AND whether they are one- or two-sided<br>*Only common tests should be described solely by name; describe more complex techniques in the Methods section.*
☒	☐	A description of all covariates tested
☒	☐	A description of any assumptions or corrections, such as tests of normality and adjustment for multiple comparisons
☐	☒	A full description of the statistical parameters including central tendency (e.g. means) or other basic estimates (e.g. regression coefficient) AND variation (e.g. standard deviation) or associated estimates of uncertainty (e.g. confidence intervals)
☒	☐	For null hypothesis testing, the test statistic (e.g. F, t, r) with confidence intervals, effect sizes, degrees of freedom and P value noted<br>*Give P values as exact values whenever suitable.*
☒	☐	For Bayesian analysis, information on the choice of priors and Markov chain Monte Carlo settings
☒	☐	For hierarchical and complex designs, identification of the appropriate level for tests and full reporting of outcomes
☒	☐	Estimates of effect sizes (e.g. Cohen's d, Pearson's r), indicating how they were calculated

Our web collection on statistics for biologists contains articles on many of the points above.

Software and code

Policy information about availability of computer code

Data collection	Illumina Nextseq 500 was used for DNA sequencing. SONY-SH800 Software was used for flow cytometry.
Data analysis	Custom scripts and notebooks are available at https://github.com/mirnylab/heterochromatin-paper. The spectral clustering pipeline is available at https://github.com/open2c/inspectro. macs2 (v2.2.7.1), UCSC tools 357, samtools (v1.7), bedtools (v2.29.2), python (v3.8.5), snakemake (v5.31.1), bwa (v0.7.17), phantompeakqualtools (v1.2.2), bioframe (v0.3.3), crossmap (v0.6.4) , pybbi (v0.3.2), cooltools (v0.5.1), cooler (v0.8.11), distiller (v0.3.3), gimmemotifs (0.17.1), picard (v2.27.4), scikit-learn (v1.1.1) , UMAP (v0.5.3), matplotlib (v3.5.2), datashader (v0.14.1), chromHMM (v1.23), plotly (v5.9.0), pomegranate (v0.4.0), polychrom (v0.1.0),

For manuscripts utilizing custom algorithms or software that are central to the research but not yet described in published literature, software must be made available to editors and reviewers. We strongly encourage code deposition in a community repository (e.g. GitHub). See the Nature Portfolio guidelines for submitting code & software for further information.

Data

Policy information about availability of data

All manuscripts must include a data availability statement. This statement should provide the following information, where applicable:

- Accession codes, unique identifiers, or web links for publicly available datasets
- A description of any restrictions on data availability
- For clinical datasets or third party data, please ensure that the statement adheres to our policy

The references and accession numbers of published data used and analyzed in this work are indicated in Supplementary Table 2. All data sets generated in this study are deposited in the NCBI Gene Expression Omnibus (GEO; http://www.ncbi.nlm.nih.gov/geo/) under the SuperSeries accession number GSE182108. SubSeries for ChIP-seq (GSE182104), Hi-C (GSE182105), Protect-seq (GSE182106), and Repli-seq (GSE182107).

Field-specific reporting

Please select the one below that is the best fit for your research. If you are not sure, read the appropriate sections before making your selection.

☒ Life sciences ☐ Behavioural & social sciences ☐ Ecological, evolutionary & environmental sciences

For a reference copy of the document with all sections, see nature.com/documents/nr-reporting-summary-flat.pdf

Life sciences study design

All studies must disclose on these points even when the disclosure is negative.

Sample size	No sample size calculations were made. All quantitative data shown is an average of at least 2 independent experiments depending on the assay as noted in Supplemental Table 1. Experiments were performed in bulk and each replicate represents millions of cells.
Data exclusions	No data were excluded from the analysis, except for Hi-C normalization. As recommended for the analysis of Hi-C data, we removed bins with low-coverage as well as contact signals between same of adjacent genomic bins.
Replication	All attempts to replicate experiments succeeded. All experiments were performed in at least duplicate.
Randomization	No randomization was done. Experiments were based on the genotype and/or condition being tested and performed in parallel.
Blinding	Investigators were not blinded to group allocation during data collection and/or analysis. Experiments were performed in parallel.

Reporting for specific materials, systems and methods

We require information from authors about some types of materials, experimental systems and methods used in many studies. Here, indicate whether each material, system or method listed is relevant to your study. If you are not sure if a list item applies to your research, read the appropriate section before selecting a response.

Materials & experimental systems

n/a	Involved in the study	
☐	☒	Antibodies
☐	☒	Eukaryotic cell lines
☒	☐	Palaeontology and archaeology
☒	☐	Animals and other organisms
☒	☐	Human research participants
☒	☐	Clinical data
☒	☐	Dual use research of concern

Methods

n/a	Involved in the study	
☐	☒	ChIP-seq
☐	☒	Flow cytometry
☒	☐	MRI-based neuroimaging

Antibodies

Antibodies used	anti-BrdU (BD:555627), anti-CTCF (CST:#3418), anti-RAD21 (abcam:ab992), anti-SMC3 (abcam:ab9263), anti-H3K27me3 (CST:#9733), anti-H3K9me2 (CST:#9753), anti-H3K9me3 (EMD:07-442), anti-HP1alpha (CST:2616)
Validation	Antibodies used in this study are commercially available and have been validated by the manufacturer. CTCF, H3K27me3, H3K9me2, HP1alpha antibodies were validated for ChIP-seq by Cell Signaling Technology. When possible datasets generated for this study were compared with those from the ENCODE project.

Eukaryotic cell lines

Policy information about cell lines

Cell line source(s)	HCT116 (ATCC CCL-247)<br>DKO (HCT116 derived, gift from B. Vogelstein to S.Pradhan, see ref. Rhee 2002)
Authentication	None of the cell lines have been authenticated.
Mycoplasma contamination	Cell lines were not tested for mycoplasma contamination but no indication of contamination was observed.
Commonly misidentified lines (See ICLAC register)	HCT116 is in the ICLAC registry. We used HCT116 verified by the ATCC because it is a well established, near-diploid, and has many available epigenetic datasets.

ChIP-seq

Data deposition

☒ Confirm that both raw and final processed data have been deposited in a public database such as GEO.

☒ Confirm that you have deposited or provided access to graph files (e.g. BED files) for the called peaks.

Data access links<br>*May remain private before publication.*	https://www.ncbi.nlm.nih.gov/geo/query/acc.cgi?acc=GSE182108
Files in database submission	See Supplemental Table 1.
Genome browser session<br>(e.g. UCSC)	HCT116 with interaction profile groups: https://resgen.io/viewer/S_dmDHLJQp66b8N9uMFQ0Q<br>Reactivated CTCF and Hi-C map: https://resgen.io/l/?d=I5UAnlnWREWRTqfvl2y5Zw<br>Compartment defect: https://resgen.io/l/?d=cd9AchRDQze6P_tuR-F4Iw

Methodology

Replicates	All ChIP-seq experiments were performed in at least 2 independent experiments depending on the assay as noted in Supplemental Table 1.
Sequencing depth	All replicate ChIP-seq experiments were sequenced to a depth >10M as a single-end read.
Antibodies	anti-BrdU (BD:555627), anti-CTCF (CST:#3418), anti-RAD21 (abcam:ab992), anti-SMC3 (abcam:ab9263), anti-H3K27me3 (CST:#9733), anti-H3K9me2 (CST:#9753), anti-H3K9me3 (EMD:07-442), anti-HP1alpha (CST:2616)
Peak calling parameters	All ChIP-seq data, including data from (Lay et al., 2015) and (Maurano et al., 2015) but excluding those obtained from the ENCODE portal, were processed following the steps of the ENCODE ChIP-seq pipeline (https://github.com/ENCODE-DCC/chip-seq-pipeline2) with slight modifications using a simplified custom snakemake workflow. Briefly, reads were mapped to hg38 using bwa mem (Li, 2013). Alignment files (BAM format) were filtered for quality and duplicates using the samtools (Li et al., 2009). Cross-correlation analysis and fragment length estimation for single-ended datasets was performed using the phantompeakqualtools package (Landt et al., 2012). Signal track (target over input) and peaks were generation was performed using MACS2 default parameters (Y. Zhang et al., 2008).
Data quality	Raw read files were assessed using fastqc prior to processing. Cross-correlation and phantom peak analysis was used to ensure data quality and estimate fragment lengths. All datasets used for peak calling received a quality tag of 1 (High) or 2 (veryHigh) from cross-correlation analysis using phantompeakqualtools.
Software	macs2 (v2.2.7.1), UCSC tools 357, samtools (v1.7), bedtools (v2.29.2), python (v3.8.5), snakemake (v5.31.1), bwa (v0.7.17), phantompeakqualtools (v1.2.2)

Flow Cytometry

Plots

Confirm that:

☒ The axis labels state the marker and fluorochrome used (e.g. CD4-FITC).

☒ The axis scales are clearly visible. Include numbers along axes only for bottom left plot of group (a 'group' is an analysis of identical markers).

☒ All plots are contour plots with outliers or pseudocolor plots.

☒ A numerical value for number of cells or percentage (with statistics) is provided.

Methodology

Sample preparation	Repli-seq was performed and analyzed as described in (Marchal et al., 2018). HCT116 cells were pulsed with 100uM BrdU for 2hrs, trypsinized, ethanol fixed, stained with propidium iodide and FACS sorted (SONY SH-800) based on DNA content (early S v late S).
Instrument	SONY-SH800
Software	SONY-SH800 Software
Cell population abundance	During two-stage Repli-seq cells are sorted based on DNA content (i.e. propidium iodide intensity). Due to the nature of unsynchronized cell populations and the imprecision of DNA content as a marker for early/late replication no further quality controls can be performed.

Gating strategy

Two gates were used to split cells in S-phase. G1 and G2 are easily identifiable and the gates were constructed to split G1/G2 evenly as the early and late fractions.

☒ Tick this box to confirm that a figure exemplifying the gating strategy is provided in the Supplementary Information.
